# Taurine Supplementation in Low-Fishmeal of Golden Pompano (*Trachinotus ovatus*) Diets: Improving Intestinal Health and Alleviation of Inflammatory Response

**DOI:** 10.3390/ani15213080

**Published:** 2025-10-23

**Authors:** Zhanzhan Wang, Hongkai Ye, Zhong Huang, Jun Wang, Yun Wang, Wei Yu, Heizhao Lin, Zhenhua Ma, Chuanpeng Zhou

**Affiliations:** 1 Key Laboratory of Aquatic Product Processing, Ministry of Agriculture and Rural Affairs, South China Sea Fisheries Research Institute, Chinese Academy of Fishery Sciences, Guangzhou 510300, China; 2 Key Laboratory of Efficient Utilization and Processing of Marine Fishery Resources of Hainan Province, Sanya Tropical Fisheries Research Institute, Sanya 572018, China

**Keywords:** *Trachinotus ovatus*, taurine, low-fishmeal diet, growth, antioxidant capacity, growth performance, intestinal health

## Abstract

This research explored the effects of supplementing taurine in a low-fishmeal diet on the growth, hepatic antioxidant capacity, muscle quality, intestinal health, and alleviation of inflammatory response of golden pompano (*Trachinotus ovatus*). Over an eight-week period, 300 juvenile fish (initial weight 9.4 ± 0.47 g) were randomly allocated into 12 net enclosures (1.0 × 1.0 × 1.5 m), with each treatment group comprising three replicate cages containing 25 specimens. The results demonstrated that an optimal taurine inclusion level of 1.0–1.5% significantly promoted growth, as evidenced by the increased weight gain rate (WGR) and specific growth rate (SGR). It also protected hepatic health by reducing alanine aminotransferase (ALT) activity and enhancing antioxidant capacity. Activation of the hepatic *Nrf2*/*Keap*-1/*HO-1* signaling pathway increased the level of antioxidant gene expression, including catalase (*CAT*) and superoxide dismutase (*SOD*). In addition, the appropriate supplementation of taurine significantly down-regulated muscle hardness-related genes (cathepsin B (*CatB*) and cathepsin L (*CatL*)) and promoted the growth and differentiation of myoblasts, thus improving muscle quality. The chymotrypsin of fish fed the A25T10 diet was significantly higher than those in other groups (*p* < 0.05). The amylase (AMY) of fish fed the A25T15 diet was significantly higher than those in other groups (*p* < 0.05). The Chao1, Shannon, and Simpson of fish fed the A25T15 diet were significantly higher than those in other groups (*p* < 0.05). *Proteobacteria* were the most abundant in group A25T10. The relative abundance of Photobacterium rose in the A25RT10 group. In this study, taurine supplementation can down-regulate the expression of intestinal pro-inflammatory factors (interleukin-1β (*IL-1β*), tumor necrosis factor-α (*TNF-α*), interleukin-8 (*IL-8*)) and up-regulate the expression of anti-inflammatory factor interleukin-10 (*IL-10*), enhance intestinal immunity, and improve intestinal digestion and absorption. Therefore, the addition of 1–1.5% taurine to low-fishmeal feeds can improve the growth performance of golden pompano.

## Simple Summary

This study investigated the effects of supplementing a low-fishmeal diet with different taurine levels on golden pompano (*Trachinotus ovatus*). This study demonstrates that adding 1.0–1.5% taurine to a low-fishmeal diet significantly improves the growth, digestive function, muscle quality, and gut health of golden pompano. This research provides a practical formula for using taurine to create effective, plant-based diets, reducing the reliance on fishmeal and lowering farming costs.

## 1. Introduction

The expansion of global aquaculture is critically constrained by the increasing scarcity and cost of fishmeal, a key resource for sustainable development [1]. This challenge has spurred significant research interest in developing low-fishmeal feeds and identifying effective nutritional additives to address potential deficiencies. China’s rapidly growing aquaculture sector has particularly exacerbated the demand for sustainable feed solutions [2]. As the predominant protein source in aquafeeds, fishmeal plays a pivotal role in determining aquaculture productivity. However, its availability is increasingly constrained by limited resources and market volatility [3]. To mitigate production costs, nutritionally dense plant-based proteins have been widely adopted as partial substitutes for fishmeal [4,5]. Nevertheless, these plant-derived alternatives often lack certain essential amino acids crucial for fish growth and development, particularly taurine [2,6]. Our previous research established that a 25% replacement of fishmeal with fermented cottonseed meal (FCSM) serves as a viable baseline, as it maintains the growth, hepatic antioxidant capacity, and intestinal health in golden pompano [7]. Building upon this foundation, the present study was designed to investigate whether taurine supplementation could further enhance the utilization of this low-fishmeal diet. Previous research has identified that dietary taurine supplementation at levels of 0.80–1.20% effectively modulates intestinal immunity by inhibiting the NF-κB pathway and ameliorating the inflammatory response [8]. To investigate whether this effective range applies to golden pompano fed a low-fishmeal diet, we selected supplementation levels of 0.5%, 1.0%, and 1.5%. *Trachinotus ovatus* (*T. ovatus*) is a commercially important species valued for its soft, fatty flesh and the absence of intermuscular spines [9].

Fishmeal serves as a crucial protein source in aquatic feeds [10], and its reduction can negatively impact the growth performance of farmed species [11]. Taurine is characterized as a sulfur-containing β-amino acid, ubiquitous in animal tissues [12]. Taurine is involved in diverse physiological functions such as osmoregulation and antioxidant activity stabilization [13]. Taurine is recognized for its ability to stimulate appetite, enhance development, facilitate digestion, bolster immunological function, and increase the antioxidant capacity [13]. Supplementation with taurine has been shown to enhance growth performance, feed efficiency, and protein utilization in several commercially important species, including juvenile catfish (*Ictalurus punetaus*) [14], rainbow trout (*Oncorhynchus mykiss*) [15], turbot (*Paralichthys olivaceus*) [16], and European seabass (*Dicentrarchus labrax*) [17]. While most fish are capable of producing taurine, supplying the taurine requirements, particularly in carnivorous species, is a challenge [18]. Studies have revealed that taurine effectively counteracts the limitations of low-fishmeal diets in aquatic animals by enhancing the growth performance [19], strengthening the hepatic antioxidant capacity [20], and promoting muscle growth and quality [6], thereby providing new insights for addressing the shortage of fishmeal resources. While taurine does not directly contribute to protein synthesis, it facilitates nutrient metabolism, improving the utilization of dietary proteins and lipids, thereby optimizing growth efficiency [21]. Studies have shown that adding an appropriate amount of taurine to low-fishmeal feeds can significantly increase the WGR, SGR, and feed efficiency of meager (*Argyrosomus regius*) [22], largemouth bass (*Micropterus salmoides*) [23], and *Litopenaeus vannamei* [24] and reduce the feed conversion ratio, ultimately improving farming profitability.

Building on the potential of FCSM as a sustainable fishmeal alternative, its application, like other plant proteins, is constrained by taurine deficiency. To bridge this gap, recent studies indicate that supplementing taurine into low-fishmeal diets might enhance development rates limited by plant protein sources and contribute to the conservation of fishmeal. Liu et al. showed that taurine supplementation, in the context of plant proteins substituting fishmeal, might enhance the development of turbot and reduce the biochemical problems in fat caused by plant protein [25]. Wei et al. showed that taurine supplementation, in the context of substituting fishmeal with plant protein, could reduce the intestine structure, immune, and oxidative problems in *Sebastes schlegeli* caused by plant proteins [26]. Current research on golden pompano nutrition has primarily focused on partial fishmeal replacement using alternative animal or plant protein sources [27,28,29]. However, limited studies have explored taurine supplementation as a strategy to enhance the utilization efficiency of low-fishmeal diets or counteract their potential negative effects in this species.

The liver serves as a crucial metabolic and detoxification organ in aquatic species [30]. A dietary fishmeal reduction has been associated with diminished hepatic antioxidant capacity and elevated oxidative stress susceptibility [31]. Taurine exhibits potent antioxidant properties, functioning through multiple mechanisms including free radical scavenging, antioxidant enzyme activation, and hepatocyte protection against oxidative damage [32]. Research indicates that adding taurine to low-fishmeal feeds can significantly increase the activities of enzymes like superoxide dismutase (SOD), catalase (CAT), and glutathione peroxidase (GSH-Px) in the liver of aquatic animals while reducing malondialdehyde (MDA) levels, thereby enhancing the hepatic antioxidant capacity and maintaining the hepatic health of juvenile black carp (*Mylopharyngodon piceus*) [33] and common carp (*Cyprinus carpio* L.) [34]. Muscle tissue constitutes the primary edible portion of farmed aquatic species, with its growth dynamics and biochemical composition directly determining its commercial value [35]. The substitution of fishmeal in aquafeeds may disrupt muscle amino acid profiles, potentially compromising both growth performance and flesh quality [36]. Taurine can promote muscle protein synthesis, inhibit protein degradation, improve muscle amino acid composition, and enhance muscle quality [37]. Research indicates that adding taurine to low-fishmeal diets can significantly increase the content of essential amino acids [38] in the muscles of aquatic animals, reduce muscle crude fat content, and improve the muscle water-holding capacity and tenderness, thereby enhancing muscle quality [6].

Therefore, building upon previous research [7] which established that a 25% replacement of fishmeal with FCSM is feasible for golden pompano, this study is designed to investigate the effects of taurine supplementation. This study includes a detailed analysis of its modulation of the gut microbiota structure, the NF-κB-mediated inflammatory response, and intestinal physical barrier function. Concurrently, the study will also assess the concomitant effects of taurine on overall growth performance, hepatic antioxidant capacity, and muscle quality. Through this comprehensive assessment, we aimed to provide a theoretical foundation for optimizing taurine use in sustainable pompano aquaculture.

## 2. Materials and Methods

### 2.1. Ethical Statement

This study complied with the ethical guidelines of the Institutional Animal Care and Use Committee at the South China Sea Fisheries Research Institute, Chinese Academy of Fishery Sciences (Guangzhou, China), following the approved protocol (SCSFRI2022-0407).

### 2.2. Diet Preparation

The nutritional composition and detailed formulation of the experimental feeds are presented in Table 1. The experimental diets were formulated as follows: MF served as the control diet without FCSM and taurine. The other three diets were formulated by replacing 25% of the fishmeal in the basal formula with FCSM and supplemented with different levels of taurine: A25T05 (0.5% taurine), A25T10 (1.0% taurine), and A25T15 (1.5% taurine). The basal protein sources consisted of fishmeal, soybean protein concentrate, corn protein powder, and soybean meal. In the experimental diets, 25% of the fishmeal in this basal formula was replaced by FCSM. Lipids were provided by fish oil and soybean lecithin. All raw materials were finely ground to pass through a 60-mesh sieve before being homogenized with aquatic oil and water. Using specialized extrusion equipment (F-26 twin-screw extruder, South China University of Technology, Guangzhou, China), the mixture was shaped into elongated strands with diameters of 2.5 mm and 3.0 mm. These strands were subsequently segmented into uniform pellets via a cutting apparatus (G-500 pelletizer, South China University of Technology, Guangzhou, China) and preserved at −20 °C in frozen storage until required for experimental use.

### 2.3. Fish Rearing and Experimental Conditions

The feeding trial was conducted in a marine pond at the Shenzhen facility of the South China Sea Fisheries Research Institute (Shenzhen, China). Prior to the trial, fish underwent a two-week acclimatization period following established laboratory protocols [39]. Following this, uniformly sized specimens (mean initial weight: 9.4 ± 0.47 g) were randomly distributed across 12 floating net enclosures (1 m × 1 m × 1.8 m; 25 fish per cage) with three replicates per dietary group. Each feed formulation was allocated randomly to triplicate cages. The fish received hand-fed meals twice daily (08:00 and 17:00) to apparent satiety over an eight-week period. Water temperature remained stable between 28.3 and 33.3 °C throughout the study. Dissolved oxygen concentrations were maintained above 6.0 mg/L, while salinity fluctuated from 20 to 25‰. Ammonia levels were controlled within 0.05–0.1 mg/L. Lighting conditions followed natural diurnal patterns.

### 2.4. Calculations

The parameters were calculated as per the following formulae:

Survival rate (SR, %) = 100 × (final number of fish)/(initial number of fish).

Weight gain rate (WGR, %) = 100 × (final body weight-initial body weight)/initial body weight.

Specific growth rate (SGR, % day^−1^) = 100 × (Ln finial individual weight − Ln initial individual weight)/number of days.

Feed conversion ratio (FCR, %) = dry feed weight (g)/(total final body weight − total initial body weight).

Protein efficiency ratio (PER, %) = fish weight gain (g)/protein intake (g)

Viscerosomatic index (VSI, %) = 100 × (viscera weight, g)/(whole body weight, g).

Hepatosomatic index (HSI, %) = 100 × (liver weight, g)/(whole body weight, g).

Condition factor (CF, g/cm^3^) = 100 × (body weight, g)/(body length, cm)^3^.

### 2.5. Sample Collection

The fish were euthanized with a 0.01% eugenol solution (Shanghai Reagent Corp., Shanghai, China) to ensure humane handling. Following sedation, all specimens within each net enclosure were counted and individually weighed. From each replicate, five fish were randomly selected for morphometric index, recording both body weight and length. For biochemical assays, portions of liver, intestinal, and muscle tissues were rapidly submerged in liquid nitrogen and later transferred to a −80 °C ultra-low temperature freezer. For the purpose of RNA extraction, tissue samples (liver, intestine, and muscle) from three fish per replicate were snap-frozen in liquid nitrogen and stored at −80 °C. Parallelly, intestinal contents were collected and preserved under the same conditions for subsequent determination of the microbial flora.

### 2.6. Parameters Measurement and Analysis

#### 2.6.1. Proximate Composition Analysis

The nutritional composition of diet formulations and raw materials was analyzed following the standardized methods established by the Association of Official Analytical Chemists (AOAC, 2005) [40]. The samples were dried in an oven at 105 °C for 72 h to determine moisture. Crude protein (N × 6.25) was determined with an automated Kjeldahl analyzer (Kjeltec 8400, FOSS, Hoganos, Sweden). Lipid levels were quantified by ether extraction using a Soxhlet apparatus (Soxtec 2055, FOSS, Hoganos, Sweden). Ash was determined using a muffle furnace (FO610C, Yamato Scientific Co., Ltd., Tokyo, Japan) at 550 °C for 5 h.

#### 2.6.2. Enzyme Activity Assay Analysis

Liver and intestinal tissues were homogenized in nine volumes (*w*/*v*) of ice-cold phosphate buffer (0.1 M, pH 7.4). The homogenate was centrifuged at 4 °C, and the supernatant was collected for assays. The following biochemical parameters were analyzed using an automated clinical chemistry analyzer (DR-200BS, Wuxi Huawei Delong Instrument Co., Ltd., Wuxi, China) following established methodology [9], including total protein (TP), alanine aminotransferase (ALT), aspartate aminotransferase (AST), catalase (CAT), glutathione peroxidase (GSH-Px), malondialdehyde (MDA), superoxide dismutase (SOD), total antioxidant capacity (T-AOC), amylase (AMY), lipase (LPS), and chymotrypsin. Enzyme activities were expressed as units per milligram of protein (U/mg prot).

#### 2.6.3. Gene Expression Level Analysis

Total RNA was extracted from liver, intestinal, and muscle tissues using the Animal Total RNA Isolation Kit (FOREGENE, Chengdu, China) according to the manufacturer’s instructions. RNA quality was confirmed by 1% agarose gel electrophoresis, and quantification was performed on a NanoDrop 2000 spectrophotometer (Thermo Fisher, Waltham, MA, USA). Subsequently, first-strand cDNA was synthesized from 1 μg of total RNA using the PrimeScript RT Reagent Kit with gDNA Eraser (Takara, Kusatsu, Japan). Synthesized cDNA aliquots were preserved at −20 °C for subsequent qPCR analysis. Gene-specific primers, including those for the reference gene β-actin (Table 2), were commercially synthesized by Sangon Biotech (Shanghai, China). Quantitative PCR was conducted on a LightCycler 480 II system (Roche, Basel, Switzerland) with S Dx PCR instrumentation (ABI, Alexandria, VA, USA). Each 12.5 μL reaction contained: 6.25 μL 2× SYBR Green Pro Taq HS Premix, 1 μL cDNA template, 0.5 μL each of forward and reverse primers, and 4.25 μL RNase-free water. Thermal cycling parameters included the following: initial denaturation (95 °C, 30 s), 40 cycles of denaturation (95 °C, 5 s), and combined annealing/extension (60 °C, 30 s), followed by melt curve analysis (60–95 °C, 0.5 °C/s increments). Target gene expression levels were normalized to β-actin and calculated using the comparative CT method (2^−ΔΔCT^) [41].

#### 2.6.4. Intestinal Microbiota Communities

Microbial genomic DNA was isolated using Hi Pure Soil DNA Kits (Magen, Guangzhou, China). The V3-V4 hypervariable region of 16S ribosomal RNA genes was amplified through polymerase chain reaction (PCR) with archaeal-specific primers Arch519 (CAGCMGCCGCGGTAA) and Arch915R (GTGCTCCCCCGCCAATTCCT). Each 50 μL PCR contained 5 μL 10× KOD Buffer, 5 μL 2.5 mM dNTPs, 1.5 μL of each primer (5 μM), 1 μL KOD Polymerase, and 100 ng DNA template. Triplicate reactions were performed for each sample. PCR products were separated by 2% agarose gel electrophoresis and purified with the AxyPrep DNA Gel Extraction Kit (Axygen Biosciences, Union City, CA, USA). Amplicon concentrations were measured using the ABI Step One Plus Real-Time PCR System (Life Technologies, Waltham, MA, USA). Equimolar amounts of purified products were pooled for paired-end sequencing (2 × 250 bp) on an Illumina platform following standard protocols. The Chao1 estimator evaluated species richness, with higher values indicating greater taxonomic diversity. The Shannon index incorporated both species abundance and evenness, where increased values reflected higher community diversity and balanced species distribution. The Simpson index inversely correlated with microbial diversity, where lower values denoted greater community heterogeneity. Bioinformatic processing was performed using the QIIME 2 pipeline (version 2023.5). Briefly, the raw sequencing data were imported, and primer sequences were trimmed using the Cutadapt plugin (version 3.4). Denoising, including quality filtering, chimera removal, and the generation of amplicon sequence variants (ASVs), was conducted with the DADA2 plugin (version 2023.5). Taxonomic assignment of representative ASVs was carried out against the SILVA 138 rRNA database using a pre-trained classifier. Alpha diversity indices (Chao1, Shannon, and Simpson) and beta diversity metrics (Weighted UniFrac distance) were calculated within QIIME 2. Principal coordinate analysis (PCoA) was performed based on the Weighted UniFrac distance matrix to visualize community structural differences. Statistical significance of group differences in beta diversity was assessed using permutational multivariate analysis of variance (PERMANOVA) with 999 permutations via the adonis2 function in the R vegan package (version 2.6–4). Visualization of community composition (bar plots) and generation of heat maps were accomplished using the R ggplot2 and pheatmap packages, respectively.

### 2.7. Statistical Analysis

Results are expressed as means ± standard error of the mean (SEM). Data analysis was conducted with SPSS Statistics 26.0 (IBM Corp., Armonk, NY, USA). Prior to analysis, all parameters were checked for normality and homogeneity of variance. Significant differences among groups (*p* < 0.05) were initially identified through one-way ANOVA. When ANOVA indicated significant effects, post hoc comparisons were made using Duncan’s multiple range test to determine specific group differences, with statistical significance set at *p* < 0.05.

## 3. Results

### 3.1. Effects of Taurine Supplementation on Growth Performance and Feed Utilization of T. ovatus

Growth performance and feed utilization of golden pompano in response to the experimental diets are shown in Table 3. The results showed that dietary taurine supplementation had a significant effect on WGR (*p* < 0.05). Among all groups, the A25T15 had the highest WGR, SGR, VSI, CF, and PER. Among all groups, the A25T05 had the highest FCR and FE. Among all groups, the HSI of MF group was the highest.

### 3.2. Effects of Taurine Supplementation on Serum Biochemical Indexes of T. ovatus

The effects of diet on serum biochemical indexes of golden pompano are shown in Table 4. The results showed that dietary taurine supplementation had no significant effect on ALT and AST (*p* > 0.05). The ALT activity of A25T10 was higher than that of other groups. The AST activity of A25T15 was lower than that of other groups.

### 3.3. Effects of Taurine Supplementation on Hepatic Antioxidant Indices of T. ovatus

The effects of diet on hepatic antioxidant indices of golden pompano are shown in Table 5. The results showed that dietary taurine supplementation had significant effects on SOD, CAT, GSH-Px, T-AOC, and MDA (*p* < 0.05). The activities of SOD and CAT in A25T10 were significantly higher than those in other groups (*p* < 0.05). The activity of GSH-Px in A25T05 and MF was significantly higher than that in other groups, and that in the A25T10 group was significantly lower than that in other groups (*p* < 0.05). The activity of T-AOC in A25T10 was significantly lower than that in other groups, and that in MF was significantly higher than that in other groups (*p* < 0.05). The MDA activity of A25T10 was significantly lower than that of other groups, and that of MF was significantly higher than that of other groups (*p* < 0.05).

### 3.4. Effects of Taurine Supplementation on the Expression of Antioxidant-Related Genes in Liver of T. ovatus

**Figure 1:**
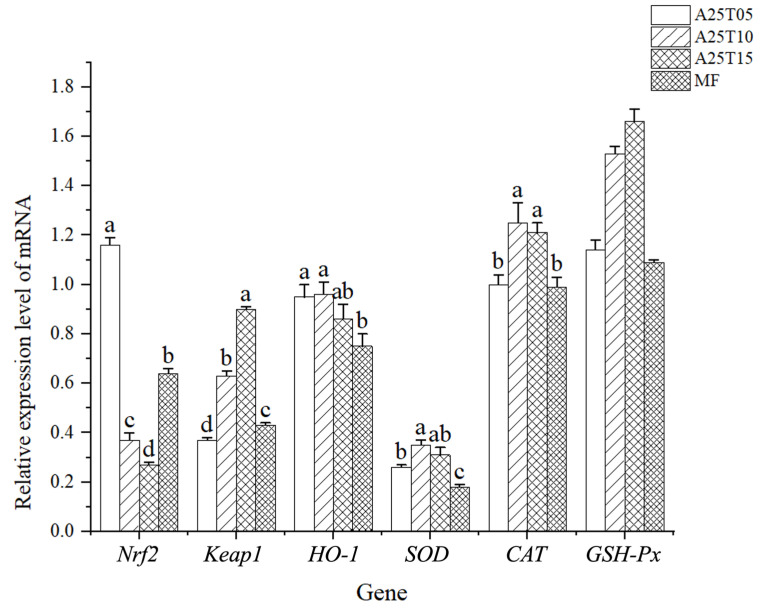
Relative mRNA expressions of antioxidant-related genes in the liver of golden pompano fed with the experimental diets. Values within a row bearing different superscript letters differ significantly (Duncan’s test, *p* < 0.05).

As shown in Figure 1, the gene expression of *Nrf2* in A25T05 was significantly higher than that in other groups (*p* < 0.05). The gene expression of *Keap-1* in A25T05 was significantly lower than that in other groups, and that in A25T15 was significantly higher than that in other groups (*p* < 0.05). The gene expression of *SOD* and *CAT* in A25T10 were significantly higher than those in other groups, and the expression of *SOD* and *CAT* in MF were significantly lower than those in other groups (*p* < 0.05). There was no significant difference in the gene expression of *GSH-Px* among the groups (*p* > 0.05).

### 3.5. Effects of Taurine Supplementation on the Expression of Growth and Quality-Related Genes in Muscle of T. ovatus

**Figure 2:**
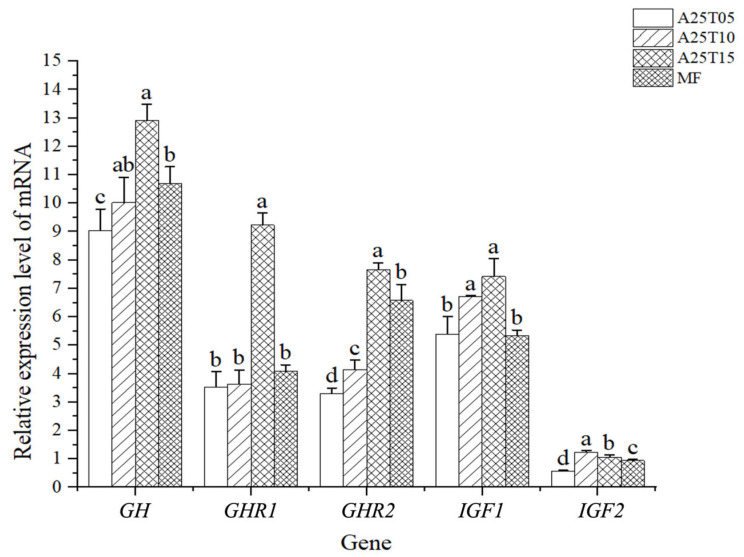
Relative mRNA expressions of growth-related genes in the muscle of golden pompano fed with the experimental diets. Significant differences (*p* < 0.05) are indicated by different superscript letters.

As shown in Figure 2, the gene expressions of *GHR1* and *GHR2* in A25T15 were significantly higher than those in other groups, and the gene expressions of *GH* and *GHR2* in A25T05 were significantly lower than those in other groups (*p* < 0.05). The gene expression of *IGF1* in MF and A25T05 was significantly lower than that in other groups (*p* < 0.05). The gene expression of *IGF2* in A25T10 was significantly higher than that in other groups, and the gene expression of *IGF2* in A25T05 was significantly lower than that in other groups (*p* < 0.05).

**Figure 3:**
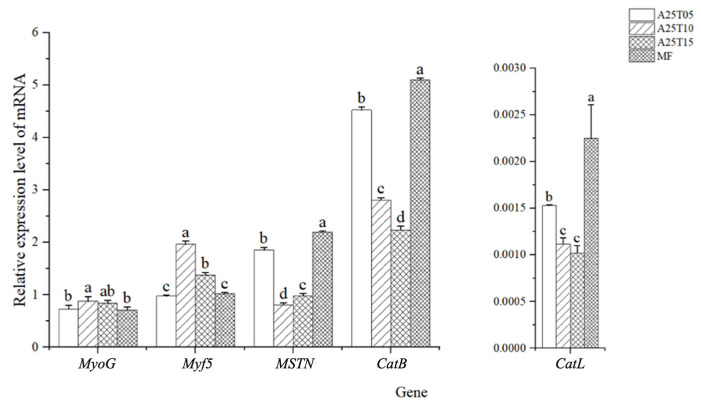
Relative mRNA expressions of quality genes in the muscle of golden pompano fed with the experimental diets. Significant differences (*p* < 0.05) are indicated by different superscript letters.

As shown in Figure 3, the gene expressions of *MyoG* and *Myf5* in A25T10 were significantly higher than that in other groups, and the gene expressions of *MyoG* and *Myf5* in MF and A25T05 were significantly lower than that in other groups (*p* < 0.05). The gene expressions of *Mstn*, *CatB*, and *CatL* in MF were significantly higher than those in other groups (*p* < 0.05). The gene expression of *CatB* in A25T15 was significantly lower than that in other groups (*p* < 0.05).

### 3.6. Effects of Taurine Supplementation on Intestinal Enzymatic Activity of T. ovatus

As shown in Table 6, the chymotrypsin of fish fed the A25T10 diet was significantly higher than those in other groups (*p* < 0.05). AMY of fish fed the A25T15 diet was significantly higher than those in other groups (*p* < 0.05). The LPS of fish fed the A25T10 diet was significantly lower than those in other groups (*p* < 0.05).

### 3.7. Effects of Taurine Supplementation Intestinal Immune-Related Gene Expression of T. ovatus

**Figure 4:**
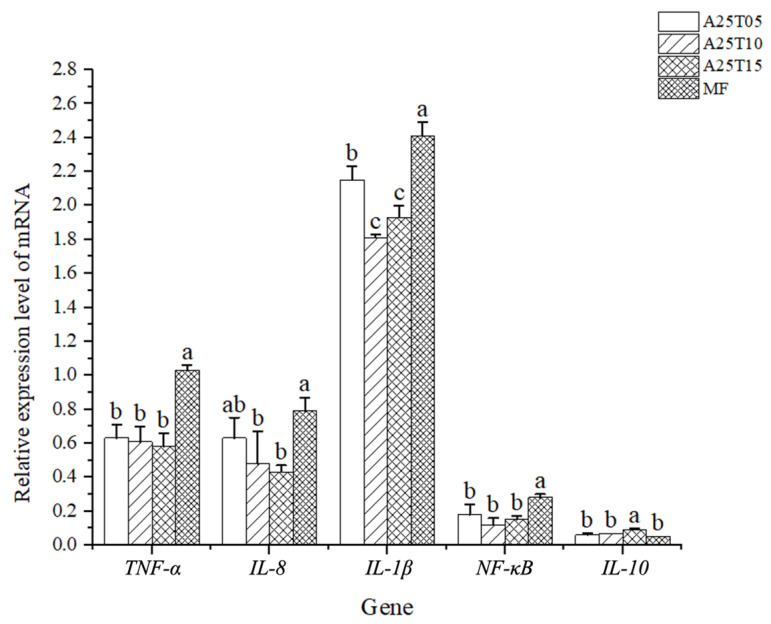
Relative mRNA expressions of immune-related genes in the intestines of golden pompano fed with the experimental diets. Significant differences (*p* < 0.05) are indicated by different superscript letters.

The gene expressions of the intestinal *NF-κB*-related signaling pathways in golden pompano after the ingestion of different experimental diets are shown in Figure 4. The relative gene expressions of *TNF-α*, *IL-8*, *IL-1β*, and *NF-κB* in fish fed with MF were significantly higher than those of the other groups (*p* < 0.05). The relative gene expression of *IL-10* in fish fed with A25T15 were significantly higher than those of the other groups (*p* < 0.05).

**Figure 5:**
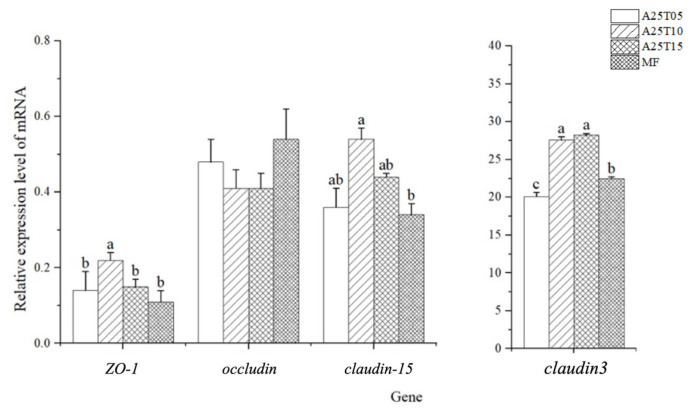
Relative mRNA expressions of physical barrier-related genes in the intestines of golden pompano fed with the experimental diets. Significant differences (*p* < 0.05) are indicated by different superscript letters.

The gene expressions of the intestinal physical barrier-related genes in golden pompano after the ingestion of different experimental diets are shown in Figure 5. None significantly affected the relative gene expression of *Occludin* (*p* > 0.05). The relative gene expressions of *ZO-1* and *Claudin-15* in fish fed with A25T10 were significantly higher than those of the other groups; those fed with MF were significantly lower than those of the other groups (*p* < 0.05). The relative gene expressions of *Claudin-3* in fish fed with A25T15 were significantly higher than those of the other groups; those fed with A25T05 were significantly lower than those of the other groups (*p* < 0.05). 

### 3.8. Effects of Taurine Supplementation on Intestinal Microbiota of T. ovatus

#### 3.8.1. Analysis of Microbial and Alpha Diversity of Intestinal Flora

**Figure 6:**
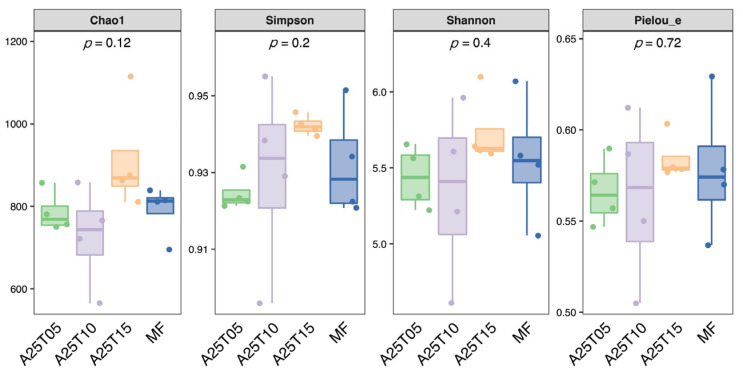
Alpha of *T. ovatus* fed different experimental diets.

As shown in Figure 6, no significant difference was observed in Chao1, Shannon, and Simpson among all treatments (*p* > 0.05). The Goods coverage of the intestinal microflora dilution of golden pompano in different treatment groups tends to be flat, with sequencing coverage of ≥99.9%. The Chao1, Shannon, and Simpson of fish fed the A25T15 diet were higher than those in other groups.

#### 3.8.2. Analysis of β Diversity of Intestinal Flora

**Figure 7:**
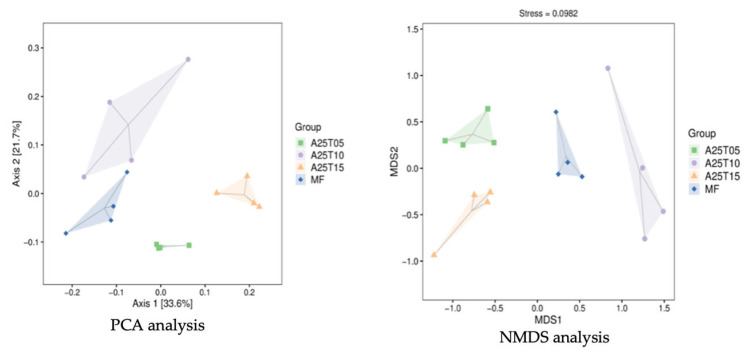
Beta of *T. ovatus* fed different experimental diets.

To assess beta diversity, PCoA was employed using Weighted UniFrac distances. The results demonstrated a clear separation of microbial communities according to dietary treatment (Figure 7).

#### 3.8.3. Analysis of Intestinal Flora Composition and Relative Abundance

**Figure 8:**
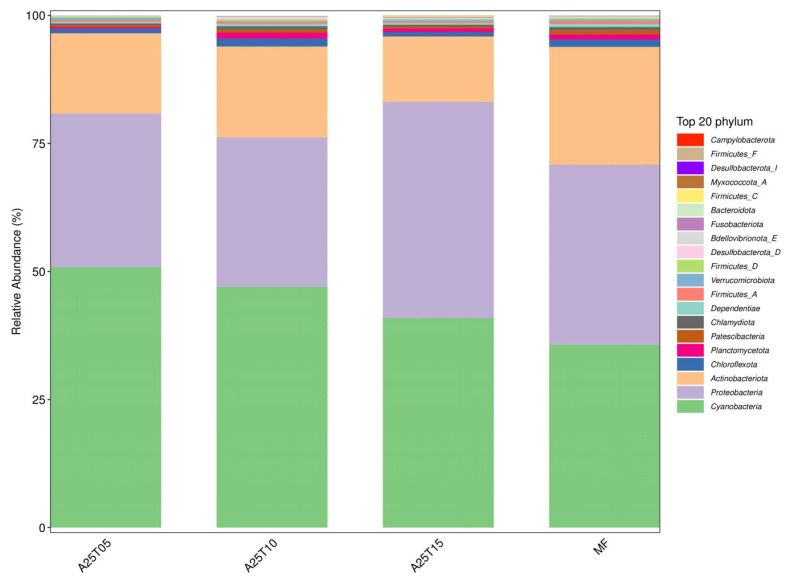
Distribution of the top 10 microbial phylum levels in the intestinal contents of *T. ovatus* fed different experimental diets.

**Figure 9:**
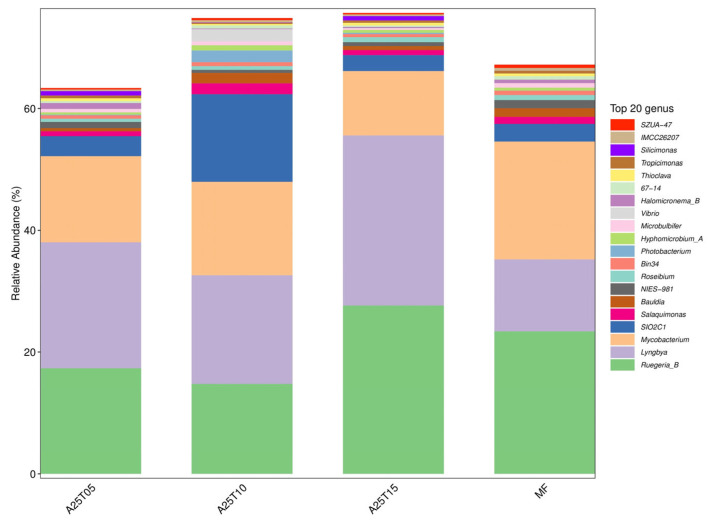
Distribution of the top 10 microbial genus levels in the intestinal contents of *T. ovatus* fed different experimental diets.

The analysis of the digestive tract contents of juvenile golden pompano entails evaluating many taxonomic classifications, including phylum, order, family, and genus. Phylum and genus were chosen to reflect typical taxonomic levels for this investigation. As shown in Figure 8, the composition of gut microflora at the phylum level consisted mainly of *Cyanobacteria*, *Proteobacteria*, and *Actinobacteriota.* At the phylum level, the gut microbiota was predominantly composed of *Cyanobacteria*, *Proteobacteria*, and *Actinobacteriota* (Figure 8). *Proteobacteria* constituted the core microbiota, with its highest relative abundance observed in the A25T10 group. The MF group contained the highest proportion of *Actinobacteriota*. At the genus level, *Ruegeria*_*B* and *Lyngbya* were the most abundant taxa in the A25T15 group (Figure 9).

#### 3.8.4. Analysis of Species Differences in Intestinal Flora

**Figure 10:**
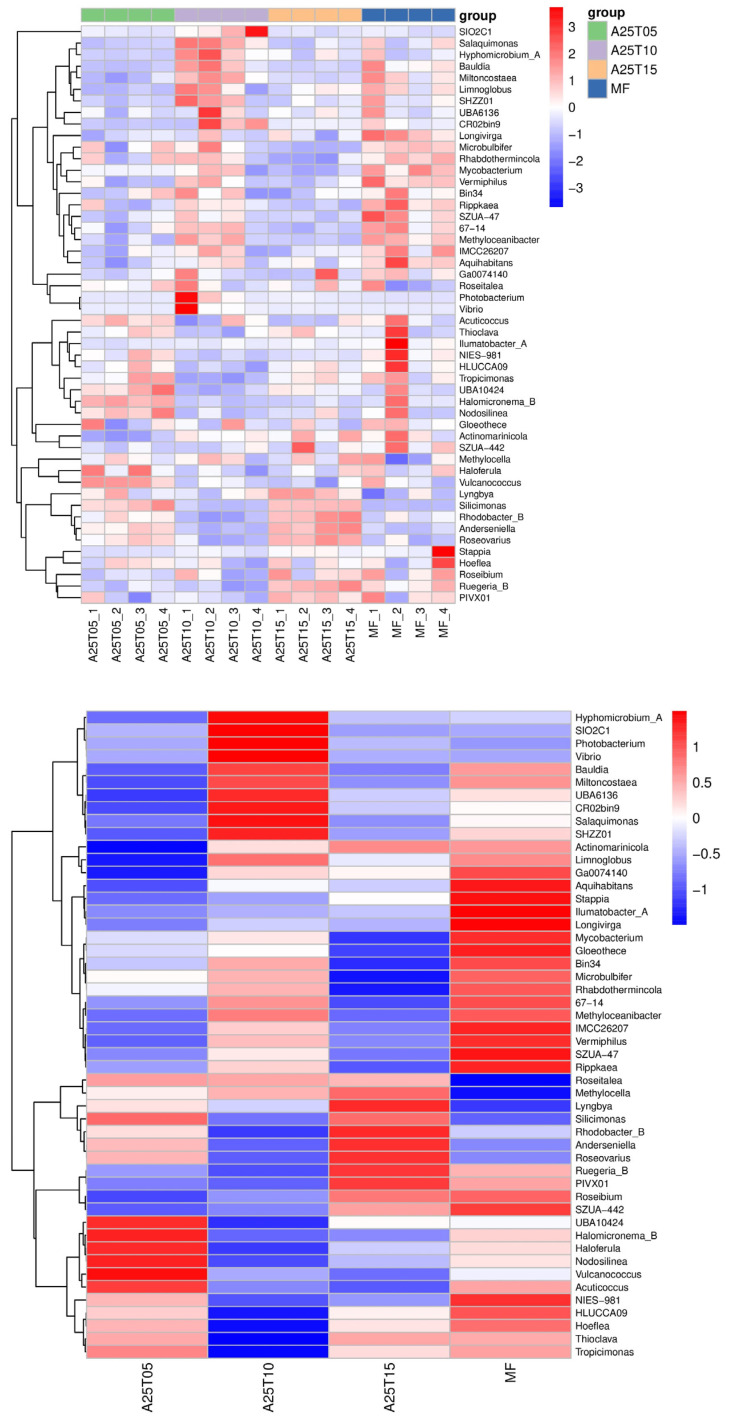
Distribution of the heat map of genus-level species composition for biclustering in the intestinal contents of *T. ovatus* fed different experimental diets.

The heat map represents an examination of prevailing species. This analysis encompasses two dimensions: Firstly, it delineates the comparative abundance of a particular dominant species across samples; secondly, it elucidates the clustering of the samples, with the clustering tree revealing the similarities and differences in the dominant species composition among them. The red blocks indicate that the genus exhibits greater abundance in that sample relative to others, whereas the blue blocks signify that the genus displays lesser abundance in that sample compared to others. The relative abundance of the five predominant bacterial groups fluctuated. As shown in Figure 10, the relative abundance of *Vulcanococcus* was elevated in the A25T05 group in comparison to the other groups. The relative abundance of Photobacterium rose in the A25RT10 group. The relative abundance of *Aquihabitans*, *Stappia llumatobacter*_A, *Longivirga*, Mycobacterium, and *Gloeothece* decreased in all other groups relative to the MF group.

## 4. Discussion

### 4.1. Effects of Taurine Supplementation on Growth Performance and Feed Utilization of T. ovatus

Taurine serves as a crucial nutrient in piscine physiology, influencing various biological functions including metabolism, immune regulation, and ontogenetic development [42,43]. Studies on catfish (*Ictalurus punetaus*) [14], Senegalese sole (*Solea senegalensis*) [44], and rainbow trout (*Oncorhynchus mykiss*) [15] demonstrate that taurine supplementation enhances growth performance by improving the feed conversion rate and protein efficiency. In the present study, golden pompano fed the low-fishmeal diet supplemented with 1.0–1.5% taurine exhibited significantly higher WGR and SGR compared to the high-fishmeal control (MF) group. This suggests that taurine inclusion is critical for achieving optimal growth in this specific low-fishmeal formulation.

### 4.2. Effects of Taurine Supplementation on Serum Biochemical Indexes of T. ovatus

Serum biochemical parameters serve as reliable biomarkers for assessing the physiological status and metabolic functions in aquatic species [45]. AST and ALT are two key transaminases, which are often used as important indicators to evaluate the degree of hepatic damage. In healthy fish, these enzymes maintain baseline concentrations in circulation. However, hepatocyte membrane integrity compromise leads to the substantial leakage of these enzymes into the bloodstream [46]. Our experimental data demonstrated that 0.5–1% taurine supplementation significantly reduced both ALT and AST activities compared to the MF group. This observation aligns with our earlier findings where a 25% fishmeal substitution with FCSM elevated the serum ALT levels, confirming its potential hepatotoxicity [39]. This reduction in serum enzyme activity is indicative of improved hepatic health and is consistent with the known hepatoprotective properties of taurine documented in other species [47].

### 4.3. Effects of Taurine Supplementation on Hepatic Antioxidant Indices of T. ovatus

Administration of taurine at appropriate levels significantly augments the antioxidant capacity in fish. Studies have shown that taurine can enhance the growth performance of Salmonids [48] and *Seriola quinqueradiata* [49]. T-AOC reflects the body’s antioxidant capacity [50]. SOD can remove excess superoxide free radicals in the body [51]. CAT can catalyze the decomposition of hydrogen peroxide in the body; GSH-PX as a key peroxidase, can catalyze the reaction of hydrogen peroxide with glutathione, thereby effectively removing hydrogen peroxide with potential hazards in organisms [7]. In this study, after adding taurine, SOD, CAT, GSH-PX, and T-AOC showed an upward trend, while MDA activity decreased, which proved that taurine significantly enhanced the antioxidant capacity of golden pompano, which was also consistent with the results of Ma et al. [52]. Furthermore, taurine demonstrates protective effects against oxidative injury in fish by modulating hepatic antioxidant enzyme activities during oxidative stress conditions.

The modulation of oxidative stress responses by taurine is primarily mediated through its regulatory effects on antioxidant enzyme systems in fish [53]. Intracellular ROS levels are primarily modulated by *Nrf2* and its inhibitor *Keap-1* with increased *Nrf2* transcription, thereby suppressing ROS accumulation. *Nrf2* can directly act on the promoter region of *HO-1* and accurately regulate its activity, thereby significantly improving the antioxidant capacity of fish in a short time [8]. In this study, taurine significantly increased the expression of *Nrf2* and *HO-1* and inhibited the expression of *Keap-1*. At the same time, it is worth noting that, in the case of adding 1–1.5% taurine, the expression levels of *SOD* and *CAT* were higher than other groups, indicating that appropriate taurine is beneficial to improve the antioxidant capacity of the hepatic of golden pompano. In the study of *Totoaba macdonaldi*, it was found that 1% taurine could improve the antioxidant capacity of fish liver and alleviate the oxidative damage caused by a low-fishmeal diet [54]. Studies on golden pompano have found that the addition of taurine to the low-fishmeal diet can activate the *Nrf2*/*Keap-1*/*HO-1* signaling pathway and enhance the antioxidant capacity of the serum and intestines of golden pompano [55]. Therefore, we believe that appropriate supplementation of taurine can alleviate the hepatic damage caused by a low-fishmeal diet and improve the antioxidant capacity of the hepatic.

### 4.4. Effects of Taurine Supplementation on the Expression of Growth and Quality-Related Genes in the Muscle of T. ovatus

Taurine supplementation influences feeding patterns in aquatic species, enhancing growth performance through stimulated appetite and increased nutrient consumption [22]. *GH* is a major gene affecting animal growth traits and has the effect of accelerating fish bone growth [56]. The role of *GH* is mainly dependent on binding to the growth hormone receptor on the cell surface and triggering a series of physiological reactions [57]. *IGF1* plays an important role in muscle growth and development [58]; *IGF2* is also a potential factor to promote growth and division and plays an important role in regulating growth, metabolism, and reproduction [59]. Our experimental data demonstrated that dietary inclusion of 1–1.5% taurine significantly up-regulated the expression of GH, GHR1, GHR2, and IGF1 genes in golden pompano. These molecular changes correspond with improved growth potential, suggesting taurine’s capacity to enhance growth-related gene expression. This is also similar to the results of previous studies on *Paralichthys olivaceus* [60].

Previous studies have found that adding 1.2% taurine to a low-fishmeal diet can significantly increase the muscle fiber density, hydroxyproline, and collagen content of turbot, thereby improving the meat quality characteristics of turbot (*Scophthalmus maximus*) [61]. Adding 2% taurine to a low-fishmeal diet can enhance the sensory characteristics of European bass (*Dicentrarchus labrax*) by increasing the hardness of fish filets and reducing drip loss [62]. It is indicated that the addition of taurine to low-fishmeal feed can improve the quality and nutritional value of aquatic animal meat. In this study, the gene expressions of *MyoG* and *Myf5* were increased, and the gene expressions of *Mstn*, *CatB*, and *CatL* were decreased when 1–1.5% taurine was supplemented into the diets, indicating that the appropriate supplementation of taurine significantly improved the muscle hardness quality of golden pompano, promoted the growth and differentiation of muscle cells, and thus improved muscle quality.

### 4.5. Effects of Taurine Supplementation on the Intestinal Health and Alleviation of Inflammatory Response of T. ovatus

Fish digestion is predominantly mediated through biochemical processes involving various digestive enzymes whose activity levels serve as reliable indicators of intestinal digestive function [9]. Studies confirm that taurine supplementation boosts key digestive enzyme (lipase and amylase) activities in the grass carp intestine, thereby enhancing its overall feed digestion capacity [63,64]. Our experimental results revealed that taurine administration significantly elevated chymotryptic and amylolytic activities in golden pompano. These findings imply that taurine may enhance intestinal digestive efficiency while mitigating the adverse effects of FCSM substitution for fishmeal on digestive function [9]. This is also similar to the results of a previous study on *Sebastes schlegeli* [26].

Fish enhance their immune function through modifying gut microbiota, preventing the intrusion of harmful microbes, and producing antimicrobial agents. Microbial composition throughout the intestines affects gut health. Probiotics in the gut decompose complicated carbohydrates and proteins, aiding in feed digestion and enhancing nutrient uptake and utilization, while encouraging growth in fish [65]. A well-balanced gut microbiota contributes substantially to host metabolism, with beneficial microbial populations promoting immune homeostasis through the competitive exclusion of pathogens and maintenance of intestinal ecological stability [66]. Notably, the alpha diversity of the gut microbiota, as measured by Chao1, Shannon, and Simpson indices, was not significantly influenced by taurine supplementation. This indicates that while taurine may have modulated the relative abundance of specific bacterial taxa, it did not cause a wholesale restructuring of the microbial community’s richness or diversity. However, the absence of significant differences in alpha diversity should be interpreted as a potential limitation. It is possible that the experimental duration was insufficient for stable microbial shifts to occur or that the sample size limited the statistical power to detect subtle but biologically relevant changes. This parallels observations showing that taurine enhances intestinal richness and diversification in aquatic species like crayfish [67] and turbot [68]. However, PCoA analysis based on Weighted UniFrac distances revealed the distinct clustering of microbial communities according to dietary treatments, suggesting that taurine specifically modulated the community composition rather than its overall diversity. This shift in community structure was accompanied by an increased relative abundance of beneficial bacteria, such as *Proteobacteria* in the A25T10 group.

Research indicates that the predominant microbiota in the fish digestive system consists primarily of *Proteobacteria*, *Actinobacteria*, *Bacteroidota*, *Firmicutes*, and other species [69]. This study showed that the highest level of Proteobacteria in the 1% taurine addition group suggests that taurine benefits the intestinal microbiota of golden pompano, confirming the results from other research [8]. The addition of taurine into low-fishmeal diets improves the intestinal flora variety and decreases the proliferation of harmful microorganisms. The NF-κB signaling pathway can regulate the transcription of a variety of cytokine mRNA, thereby affecting the body’s inflammatory response [28]. The inflammatory response in fish is orchestrated by a balance between pro-inflammatory factors (e.g., *IL-1β*, *IL-8*, and *TNF-α*) and anti-inflammatory factors (e.g., *IL-10*) [8]. In this study, the gene expression of intestinal *IL-1β*, *IL-8*, and *TNF-α* were down-regulated, and the gene expression of *IL-10* was up-regulated in the case of dietary supplementation with 1–1.5% taurine, indicating that the appropriate supplementation of taurine can enhance the intestinal resistance to inflammation. Our findings on the inflammatory effects of taurine corroborate those observed in *Ictalurus punctatus* [53].

The intestinal health of fish is closely related to the intestinal immune barrier function and structural barrier function [70]. The health of the fish intestine is not only related to the immunity of the intestine but also the structural integrity [71]. In this study, the gene expression of *ZO-1*, *Claudin-3*, and *Claudin-15* were increased when the diet was supplemented with 1–1.5% taurine, indicating that the appropriate supplementation of taurine could enhance the physical barrier function of the intestinal tract. This is also similar to the previous research results on *Micropterus salmoides* [72].

## 5. Conclusions

In conclusion, this study identified that supplementing 1.0–1.5% taurine in a low-fishmeal diet (with 25% fishmeal replaced by fermented cottonseed meal) resulted in the growth performances and physiological conditions in golden pompano that were equivalent to a high-fishmeal control. The improved growth performance can be attributed to taurine’s role in primarily enhancing intestinal health by improving digestive enzyme activity and reinforcing the physical barrier, which likely facilitated better nutrient absorption. Concurrently, it systemically protected the organism by increasing the hepatic antioxidant capacity through the *Nrf2* pathway and reducing inflammation, thereby fostering a more favorable physiological state for growth.

## Figures and Tables

**Table 1:** Formulation and nutrient levels of the experimental diets (% dry matter).

Ingredients	Diets			
	A25T05	A25T10	A25T15	MF
Fishmeal	18.75	18.75	18.75	25
Soy protein concentrate	12	12	12	12
Corn protein powder	10	10	10	10
Soybean meal	8	8	8	8
Fermented cottonseed meal	6.19	6.19	6.19	0
Corn starch	12.65	12.65	12.65	14.44
Porcine blood cell protein powder	8	8	8	8
Beer yeast powder	2	2	2	2
Fish oil	4.9	4.9	4.9	4.64
Soybean oil	4.9	4.9	4.9	4.64
Vitamin and mineral premix ^1^	1	1	1	1
Ca(H_2_PO_4_)_2_	0.5	0.5	0.5	0.5
Choline chloride	0.5	0.5	0.5	0.5
Lecithin	1	1	1	1
Microcrystalline cellulose	7.54	7.04	6.54	6.71
Betaine	0.5	0.5	0.5	0.5
Lysine	0.31	0.31	0.31	0.16
Methionine	0.15	0.15	0.15	0.09
Glycine	0.61	0.61	0.61	0.82
Taurine	0.5	1	1.5	0
Total	100	100	100	100
Nutrient levels ^2^				
Ash	5.05	5.05	5.05	5.67
Crude protein	42.34	42.34	42.34	42.35
Crude lipid	12.47	12.47	12.47	12.48

^1^ Vitamin and mineral premix provided by Shenzhen Jingji Zhinong Times Co., Ltd. (Shenzhen, China, mg kg^−1^ diet) and was composed of the following (per kg of diet): Vitamin A (≥450,000 IU), Vitamin B1 (≥1000 mg), Vitamin B2 (≥1000 mg), Vitamin B6 (≥1500 mg), Vitamin B12 (≥5 mg), Vitamin K3 (≥800 mg), inositol (≥12,000 mg), D-Pantothenic acid (≥3500 mg), nicotinic acid (≥2000 mg), folic acid (≥500 mg), D-Biotin (≥5 mg), Vitamin D3 (300,000–400,000 IU), Vitamin E (≥8000 IU), Na_2_SeO_3_ (20 mg), CuSO_4_·5H_2_O (24 mg), FeSO_4_·H_2_O (266.65 mg), ZnSO_4_·H_2_O (100 mg), MnSO_4_·H_2_O (120 mg), Ca(IO_3_)_2_ (50 mg), CoSO_4_·7H_2_O (10 mg), Mg (20 g), and zeolite (4380.55 mg). ^2^ Theoretical values.

**Table 2:** The primers for real-time fluorescence quantification PCR.

Gene	Sequence (5′-3′)	Reference
*GH*-qF	GCCAGTCAGGACGGAG	[7]
*GH*-qR	AGGAGGCGGGGCTACA	
*GHR1*-qF	GGTGGAGTTCATTGAGGTGGAT	[7]
*GHR1*-qR	TGGTGGCTGACAGGTTGG	
*GHR2*-qF	CACCACCTCTACCTCCTCTG	[7]
*GHR2*-qR	CCCTCTTCGGCGTTCATA	
*IGF1*-qF	GACGCTTACAGGAGGAGAA	[7]
*IGF1*-qR	GCTGCTGGATGTGTTCAC	
*IGF2*-qF	CTGTGACCTCAACCTGCT	[7]
*IGF2*-qR	CTCTGCCACTCCTCGTATT	
*β-actin*-qF	TACGAGCTGCCTGACGGACA	[7]
*β-actin*-qR	GGCTGTGATCTCCTTCTGCA	
*myoG*-qF	AACCAGAGGCTGCCCAAGG	[7]
*myoG*-qR	GCTGTCCCGTCTCAGTGTCC	
*myf5*-qF	AAGAACGAGAGTTTGGGCGA	[7]
*myf5*-qR	AGGACGTGGTATATGGGCCT	
*MSTN*-qF	GACGGGAACAGGCACATACG	[7]
*MSTN*-qR	GCAGCCACACGGTCAACACT	
*CatL*-qF	CCACTGGCACCTCTGCAAGA	[7]
*CatL*-qR	GCCCGTAGCACTGTTTGCCC	
*CatB*-qF	TCTGCCTGGGACTTCTGGACCA	[7]
*CatB*-qR	ACACTTGAGGACGCACTGAG	
*Nrf2*-qF	TTGCCTGGACACAACTGCTGTTAC	[7]
*Nrf2*-qR	TCTGTGACGGTGGCAGTGGAC	
*Keap1*-qF	CAGATAGACAGCGTGGTGAAGGC	[7]
*Keap1*-qR	GACAGTGAGACAGGTTGAAGAACTCC	
*HO-1*-qF	AGAAGATTCAGACAGCAGCAGAACAG	[7]
*HO-1*-qR	TCATACAGCGAGCACAGGAGGAG	
*SOD*-qF	CCTCATCCCCCTGCTTGGTA	[7]
*SOD*-qR	CCAGGGAGGGATGAGAGGTG	
*GSH-Px*-qF	GCTGAGAGGCTGGTGCAAGTG	[7]
*GSH-Px*-qR	TTCAAGCGTTACAGCAGGAGGTTC	
*CAT*-qF	GGATGGACAGCCTTCAAGTTCTCG	[7]
*CAT*-qR	TGGACCGTTACAACAGTGCAGATG	
*il-1β*-qF	CGGACTCGAACGTGGTCACATTC	[9]
*il-1β*-qR	AATATGGAAGGCAACCGTGCTCAG	
*IL-8*-qF	TGCATCACCACGGTGAAAAA	[9]
*IL-8*-qR	GCATCAGGGTCCAGACAAATC	
*TNF-α*-qF	CGCAATCGTAAAGAGTCCCA	[9]
*TNF-α*-qR	AAGTCACAGTCGGCGAAATG	
*il-10*-qF	CTCCAGACAGAAGACTCCAGCA	[9]
*il-10*-qR	GGAATCCCTCCACAAAACGAC	
*NF-κB*-qF	TGCGACAAAGTCCAGAAAGAT	[9]
*NF-κB*-qR	CTGAGGGTGGTAGGTGAAGGG	
*ZO-1*-qF	TTTGTGGCAGGAGTTCT	[9]
*ZO-1*-qR	TTCTTGTTGGGGATGAT	
*Occludin*-qF	TACGCCTACAAGACCCGCA	[9]
*Occludin*-qR	CACCGCTCTCTCTGATAAA	
*Claudin-3*-qF	CTCCTCTGCTGCTCCTGTCC	[9]
*Claudin-3*-qR	CGTAGTCTTTCCTTTCTAACCCTG	
*Claudin-15*-qF	AAGGTATGAAATAGGAGAAGGGC	[9]
*Claudin-15*-qR	TGGTTTGATAAGGCAGAGGGTA	

**Table 3:** Growth performance and feed utilization of *T. ovatus* fed different experimental diets.

Item	Diets			
	A25T05	A25T10	A25T15	MF
WGR (%)	245.48 ± 12.91 ^b^	304.11 ± 21.18 ^ab^	380.46 ± 17.99 ^a^	286.48 ± 89.39 ^ab^
SGR (%)	2.22 ± 0.07	2.49 ± 0.09	2.80 ± 0.07	2.38 ± 0.45
FCR	2.65 ± 0.22	2.30 ± 0.10	1.86 ± 0.05	2.57 ± 0.91
FE	1.3 ± 0.06	1.27 ± 0.03	1.1 ± 0.00	1.27 ± 0.12
PER	0.93 ± 0.03	1.1 ± 0.06	1.33 ± 0.03	1.03 ± 0.22
VSI (%)	1.01 ± 0.10	1.02 ± 0.31	1.22 ± 0.27	1.08 ± 0.39
HSI (%)	7.49 ± 0.93	7.61 ± 1.50	7.56 ± 1.04	7.96 ± 0.99
CF (%)	1.70 ± 0.07	1.76 ± 0.02	1.79 ± 0.08	1.76 ± 0.19

The data include triplicate means. Means in the same row that have distinct superscript letters are substantially different, as determined by Duncan’s test (*p* < 0.05).

**Table 4:** Serum indices of *T. ovatus* fed different experimental diets.

Enzyme	Diets			
	A25T05	A25T10	A25T15	MF
ALT(U/L)	2.52 ± 0.48	3.31 ± 0.49	2.83 ± 0.9	3.13 ± 0.84
AST(U/L)	35.38 ± 8.25	30.68 ± 4.45	27.81 ± 2.06	35.9 ± 2.8

**Table 5:** Hepatic antioxidant indices of *T. ovatus* fed different experimental diets.

Enzyme	Diets			
	A25T05	A25T10	A25T15	MF
SOD (U/mg)	17.48 ± 0.65 ^b^	18.8 ± 0.43 ^a^	8.1 ± 0.55 ^d^	11.57 ± 0.34 ^c^
CAT/(U/mg)	5.85 ± 0.20 ^d^	20.34 ± 0.38 ^a^	15.6 ± 0.74 ^b^	11.45 ± 0.23 ^c^
GSH-Px/(U/mg)	0.0345 ± 0.00125 ^a^	0.0234 ± 0.00085 ^c^	0.031 ± 0.0000 ^b^	0.0339 ± 0.00114 ^a^
T-AOC/(U/mg)	0.0796 ± 0.00201 ^c^	0.0614 ± 0.00129 ^d^	0.0862 ± 0.0016 ^b^	0.0922 ± 0.00169 ^a^
MDA (nmol/mg)	1.9 ± 0.05 ^d^	2.6 ± 0.03 ^c^	2.74 ± 0.09 ^b^	3.62 ± 0.05 ^a^

The data include triplicate means. Means in the same row that have distinct superscript letters are substantially different, as determined by Duncan’s test (*p* < 0.05).

**Table 6:** Intestinal digestive enzymes of *T. ovatus* fed different experimental diets.

Enzyme	Diets			
	A25T05	A25T10	A25T15	MF
Chymotrypsin (U/mg)	0.0436 ± 0.00225 ^d^	0.0834 ± 0.0026 ^a^	0.0615 ± 0.00122 ^b^	0.0538 ± 0.00127 ^c^
AMY(U/mg)	0.0084 ± 0.0003 ^b^	0.0081 ± 0.00012 ^b^	0.0094 ± 0.00026 ^a^	0.0073 ± 0.0001 ^c^
LPS (U/mg)	0.0415 ± 0.00146 ^b^	0.028 ± 0.00015 ^d^	0.0383 ± 0.00153 ^c^	0.0445 ± 0.00093 ^a^

The data include triplicate means. Means in the same row that have distinct superscript letters are substantially different, as determined by Duncan’s test (*p* < 0.05).

## Data Availability

Data for this research article were available from the corresponding author by reasonable request.
